# The Eyesight of School Children

**Published:** 1896-07-25

**Authors:** 


The Hospital

An Institutional Journal of

The Medical Sciences and Hospital Administration, with

AN EXTRA NURSING SUPPLEMENT.

Vol. XX.—No. 513.] [July 25, 1896.

### The Eyesight of School Children.

A report, signed by Mr. Brudenell Carter, acting for a specially-appointed Committee of the Education Department, gives the results of a carefully-conducted investigation into the condition of the eyesight of more than 8,000 children attending our public elementary schools. The importance of the subject is to be measured by the facts that the eight thousand children thus examined may be taken as typically representative of their class; and that the conditions of eyesight found in them will in all probability be found in all the children attending elementary schools throughout the kingdom, at any rate in large towns, and no doubt more or less similar conditions will be discovered to be characteristic of the eyesight of town-bred children of all classes. What then we have to consider is not the mere circumstance that of 8,000 London children more than half have defective eyesight, but that more than half of all the children of the lower middle class throughout the large towns of the kingdom are in that condition. With circumstances of such magnitude, the State may well be called upon to deal promptly; and if medicine has anything to say whereby such conditions of eyesight may be modified, or altogether "improved away" among our poorer children, she should say it with all reasonable authority and decision.

To be strictly precise in figures, Mr. Brudenell Carter found that of the 8,125 children only 3,181, or 39·15 per cent., were endowed with sight of the "first order," that is, with normal vision in both eyes. Of the remaining 4,944, as many as 1,016, or 12·5 per cent., though of normal vision in the right eye were subnormal in the left; 700, or 8·6 per cent., were normal in the left but subnormal in the right, whilst the enormous total of 3,228, or 39·7 per cent., showed subnormal vision in both eyes. Carrying the investigation further, Mr. Brudenell Carter measured the vision in 1,448 children previously reported as "subnormal," with the result that 406 were found to be endowed with two-thirds of normal visual power in both eyes, 140 with one-half, and 197 with less than half in both eyes. From all of which it may be seen that, broadly speaking, many more than half of our children suffer from defective eyesight, whilst a very large number have sight of the poorest possible quality; and must, *ipso facto*, be disqualified for any but the very roughest and commonest of avocations.

The question which parents and school managers will ask is, whether such an investigation as Mr. Brudenell Carter has conducted may be expected to bear fruit of a practical kind in the improvement of the eyesight? Undoubtedly it may; and Mr. Carter, who is a physiologist as well as a competent oculist, puts his finger on the exact cause of the mischief, and therefore on the exact spot at which we must begin in our efforts at treatment and cure. The retina, as he tells us, is not adequately cultivated in London school children. But, since no kind of perfection can be obtained in any structure of the body without the exercise and training of that structure, neither can the retina be developed to any standard of perfection except by similar training. This is decidedly interesting, and though in no sense new to the physiologist, it will be felt to be somewhat novel by school managers. They have not been accustomed to think of the retina in this way as a nerve expansion which needs training exactly as the nerves of the tea-taster's tongue need training, or the nerve-bulbs of the violinist's fingers.

Mr. Brudenell Carter is very decided, not to say enthusiastic, on this question of the training of the retina. It had been thought, before this investigation took place, that the schools and the general conditions of school life, in the metropolis and elsewhere, had probably brought about the defective eyesight of which school managers have recently become so conscious. Not so decides Mr. Carter. "I feel certain," says he, "that the subnormality of vision in school children must be attributed chiefly, if not entirely, to the conditions of their lives and surroundings, although not to those of their schools." London children, he tells us, "see the other side of the street in which they live, with the carts and omnibuses of the thoroughfares," and they see little else. "They scarcely ever have the visual attention strongly directed to any object which it is difficult to see." In marked contrast with this is the case of children bred and reared in country villages. A country child, Mr. Carter tells us, "has an expanse of landscape before him, presenting numerous objects under visual angles rendered small by distance. He finds, moreover, attractions in every hedgerow, flowers, insects, birds' nests, many of them disguised by their resemblance in colour to their surroundings, and requiring close scrutiny in order that they may be distinguished." In this way the retina of the country lad is "trained," and Mr. Carter expresses the opinion that the sight of the average village boy and girl, if examined, would be found to be as much above the normal standard as that of the town child is undoubtedly below it.

In this investigation we have an illustration of the kind of work, altogether apart from the mere art of healing, which scientific medicine and physiology are doing for civilisation. What is to be hoped is that Parliament, municipalities, and the public generally will give such work wide and generous recognition.

